# Influence of Geometric and Manufacturing Parameters on the Compressive Behavior of 3D Printed Polymer Lattice Structures

**DOI:** 10.3390/ma14061462

**Published:** 2021-03-17

**Authors:** Rafael Guerra Silva, Cristóbal Salinas Estay, Gustavo Morales Pavez, Jorge Zahr Viñuela, María Josefina Torres

**Affiliations:** School of Mechanical Engineering, Pontificia Universidad Católica de Valparaíso, Quilpué 2430000, Chile; cristobal.salinas.e@mail.pucv.cl (C.S.E.); gustavo.morales@pucv.cl (G.M.P.); jorge.zahr@pucv.cl (J.Z.V.); josefina.torres@pucv.cl (M.J.T.)

**Keywords:** lattices, cellular materials, fused deposition modeling, additive manufacturing

## Abstract

Fused deposition modeling represents a flexible and relatively inexpensive alternative for the production of custom-made polymer lattices. However, its limited accuracy and resolution lead to geometric irregularities and poor mechanical properties when compared with the digital design. Although the link between geometric features and mechanical properties of lattices has been studied extensively, the role of manufacturing parameters has received little attention. Additionally, as the size of cells/struts nears the accuracy limit of the manufacturing process, the interaction between geometry and manufacturing parameters could be decisive. Hence, the influence of three geometric and two manufacturing parameters on the mechanical behavior was evaluated using a fractional factorial design of experiments. The compressive behavior of two miniature lattice structures, the truncated octahedron and cubic diamond, was evaluated, and multilinear regression models for the elastic modulus and plateau stress were developed. Cell size, unit cell type, and strut diameter had the largest impact on the mechanical properties, while the influence of feedstock material and layer thickness was very limited. Models based on factorial design, although limited in scope, could be an effective tool for the design of customized lattice structures.

## 1. Introduction

Additive manufacturing (AM) opens up new opportunities for the development of novel materials, as they are capable of generating complex custom-made lattice structures in three-dimensions with precise control over the size and shape of both cells and struts, and the overall topology of the structure [1]. Applications of lattices include bioengineering devices [2], acoustics [3], thermal management [4,5,6], and energy absorption in personal and sports protective equipment [7].

In recent years, the design, simulation and fabrication of lattices built via AM have attracted attention, and different methods have been proposed to predict the mechanical performance of lattice structures [8]. However, some design issues like the selection of the unit cell type and its optimal parameters are still open questions [9].

Thus, the characterization of a multitude of lattice configurations manufactured using diverse AM methods such as fused deposition modeling [7,10,11,12,13], stereolithography [14,15,16], jet fusion [17,18,19], and selective laser sintering [20,21] is currently a very active topic in research, in addition to the development of diverse analytical and numerical methods for the design of lattice structures [22,23,24,25].

The influence of diverse geometric parameters (unit cell type, cell size, cell orientation, strut thickness, strut cross-section, etc.) on the mechanical properties of lattice structures has been investigated by analytical, numerical, and experimental techniques [7,26,27,28,29,30,31]. For instance, scaling law models have been used to model the relationship between strut diameter, cell size, and mechanical properties [22,28], and have been validated experimentally [7,29,30,31].

Experimental studies of FDM-fabricated lattices in particular have been scarce, as alternative AM methods offer better accuracy and quality [18,32]. However, the use of FDM could be effective in the production of functional self-supporting miniature lattice structures with a wide variety of unit cell types [13].

A handful of studies have explored the effect of geometric features in FDM-fabricated lattices. Al Rifaie et al. [12] compared the compressive behavior of four types of body-centered cubic lattices manufactured using FDM. Stiffness, failure loads, and energy absorption capacity under quasi-static compression were reported, recognizing the influence of vertical support struts on the mechanical properties of lattice structures. Similarly, Karamooz Ravari et al. [10] manufactured and tested body-centered cubic lattices. The results of compressive tests were compared to analytical and finite element models, confirming that strut irregularities related to the manufacturing process lead to lower mechanical properties of polymer lattice structures built by FDM compared to analytical and numerical predictions. Rossiter et al. [7] evaluated the effect of five geometric design variables (cell size, strut cross-section shape and area, cell arrangement, and strut filleting), on the compressive behavior of truncated octahedron lattices manufactured by FDM. The factorial design of experiments was used to study the influence of the variables and their interactions, evidencing that strut thickness and cell width had the largest effect on the plateau stress and energy absorption capacity. Guerra et al. [13] characterized the compressive behavior of diverse miniature lattice structures fabricated by FDM. Although the role of both hardware and software in the mechanical strength of the lattices was discussed, the influence of specific manufacturing parameters on the mechanical properties was not explored.

One distinctive feature of AM-fabricated miniature lattices is the presence of irregularities in struts, which affect their effective mechanical properties [23,33,34,35]. Although several studies have considered methods to predict the effect of strut irregularities on the mechanical response of lattices [30,36,37], they are considered an unavoidable consequence of the AM processes. Although irregularities have been loosely connected to process parameters in AM-fabricated metal lattices [38] and some manufacturability issues regarding layer thickness have been discussed [13,39], little attention has been given to a possible relationship between the AM process parameters and these irregularities. Furthermore, research on the effects of AM process parameters on the mechanical response of lattice structure has been scarce or addressed only indirectly. Harrysson et al. [38] considered the staircase effect in lattices manufactured by electron-beam melting but did not establish a relationship between AM process parameters and mechanical properties of lattices. Yan et al. [29] discussed the causes of irregular struts in metal lattices fabricated using selective laser melting, suggesting that laser melting depth, a process parameter that determines the depth of laser melting into the powder, could play a role in the overall quality of the lattice structure. Cahill et al. [40] evaluated the effect of part orientation in the SLS fabrication of polyamide miniature lattices (cell size ~2.5 mm), reporting a significantly larger compressive strength in the z-direction.

Regarding FDM-fabricated lattices, research is also very limited. Gautam et al. [11] evaluated the compressive performance of large FDM-fabricated acrylonitrile butadiene styrene (ABS) single Kagome truss unit cells (cell size 35 mm), reporting a variation of approximately 20% in the compressive strength of the lattices when either build orientation or surface roughness were modified. No research has been presented dealing with the effects of AM process parameters on the mechanical properties of FDM-fabricated miniature lattices (cell size < 5 mm/strut diameter < 1 mm).

On the other hand, the influence of FDM process parameters on the mechanical properties of solid polymer specimens has been extensively studied [41,42,43,44,45,46,47]. The most common FDM process parameters that can be controlled include layer thickness, build orientation, raster angle, and infill density. Layer thickness is defined as the height of deposited slices. Build orientation is defined according to the position of the part (horizontally or vertically) in the printing bed [45]. Raster width is the thickness of the extrudate deposited by the nozzle. Raster angle (or raster orientation) refers to the direction of the raster relative to the bed [48]. Other FDM process parameters include nozzle diameter, flow rate, deposition speed, raster pattern, air gaps (raster to raster, perimeter to raster), number of contours/perimeters (contour width), top thickness, and bottom thickness [45].

The most important parameters influencing the mechanical properties of FDM parts that should be considered include raster-to-raster air gap, raster angle, layer thickness, infill density, and build orientation [41]. Other factors such as build orientation and layer thickness also affect the strength and durability of components produced by FDM [41]. Similarly, process parameters like air gap and raster orientation significantly affect the strength of FDM processed parts, while other parameters such as raster width and extrusion temperature have little effect [48]. The extrusion temperature does not affect the mechanical properties significantly, as long as the filament is heated enough to be extruded properly [49]. On the other hand, variations in the envelope temperature–defined as the environmental temperature surrounding the FDM process–could have strong effects on the mechanical properties of parts [48].

In the present work, the compressive behavior of two miniature lattice structures, the truncated octahedron and cubic diamond, is analyzed to evaluate the effect of geometric and manufacturing parameters on their mechanical properties. Additionally, multilinear regression models derived from experimental data are presented for the prediction of the plateau stress and elastic modulus.

## 2. Materials and Methods

### 2.1. Experimental Design

Several manufacturing parameters were excluded early in the design stage, based on their lesser effect on the mechanical properties of FDM-fabricated parts, as reported in other studies, such as raster width. Other parameters were excluded for different practical reasons: extrusion and envelope temperature were excluded, as optimal values are commonly given by feedstock manufacturers; nozzle diameter was set to 0.4 mm, due to availability issues; neither printing speed nor air gap can be modified.

Parameters such as raster width, raster-to-raster air gap, raster angle, and infill density were not included, as the cross-section area of struts is too small in miniature lattices to allow for an infill pattern. As for build orientation, variations in AM-fabricated miniature lattices in build orientation only cause small changes in mechanical properties (~5%) [50].

On the other hand, layer thickness could be of great importance, as it determines the dimensional accuracy of parts [51,52]. This could be of special interest in miniature lattice structures, as the dimension of the struts is relatively small and the staircase effect could have a negative impact [38,39]: the interaction between strut orientation and layer thickness affects the accuracy of struts in AM and could cause significant changes in mechanical properties, as strut thickness must grow larger if the angle between the strut and the horizontal plane increases to secure adequate adhesion between layers. The layer thickness determines the height of the step: the lower the layer thickness, the smaller the staircase effect on the prototype [53].

Finally, five parameters that could influence the mechanical properties of miniature lattice structures were selected. Cell size and unit cell type, and strut diameter define the overall strength of lattices; feedstock material and layer thickness were taken into consideration as both the material of struts and the staircase effect could influence the mechanical properties of lattice structures.

A two-level fractional factorial design was used, with a low and a high level for each parameter (Table 1). A 2^5-1^ design was used, to reduce the number of tests and explore the interaction between parameters [54]. Two-level fractional factorial designs are widely used in the early stages of experiments to screen important factors from a large number of potential factors [55]. The software Minitab (version 19, Minitab LLC, State College, Pennsylvania, USA) was used to set up the design.

The levels for the factorial design were defined according to the limits set by the dimension of the unit cells and the manufacturing process while maximizing the difference between them [13].

Two different unit cell types were selected for the study: cubic diamond, designed using the software nTopology (nTopology Inc., New York, NY, USA) [56], and truncated octahedron, designed using Autodesk Inventor. Both unit cell types have been extensively studied, and their potential for energy absorption and biomedical applications has been evaluated [7,24,33,57,58,59]. Truncated octahedron lattice structures show high compressive strength compared to other structures [58], while the cubic diamond unit cell typically has a lower compressive strength [24,57]. As for cell size and strut thickness, the minimum feasible dimensions in FDM were selected as lower bound values, 1 mm and 5 mm, respectively. Strut cross-section was defined as circular in the digital model. Two different printing materials were evaluated, acrylonitrile butadiene styrene (ABS) and polycarbonate (PC), which have similar density and elastic modulus, but different compressive yield strength, a parameter that determines the plateau stress in cellular structures [22]. A comparison of the mechanical properties of ABS and PC is presented in Table 2.

The low level of layer thickness was set to the minimum value possible with the printer, while the high level was set to the mid-range value (0.15–0.45 mm). Both values do offer enough printing resolution to correctly generate the smallest features in the structure.

The 16 proposed configurations to be tested and their characteristics are listed in Table 3. For error estimation, two specimens of every configuration were manufactured and tested under uniaxial compression, totaling 32 samples/tests.

### 2.2. Manufacturing of Specimens

Test specimens with length scales of 35 mm × 35 mm × 35 mm, similar to the specimen size reported by other researchers [24,25], were prepared using FDM technology, employing a desktop FDM machine (Up mini 2, Tiertime, Beijing, China). The slicing software Up Studio v2.6 (Tiertime, Beijing, China) was used to process the STL files. No support was required during the manufacturing process, as the selected lattice structures were self-supporting. No special methods were required to secure adhesion to the printing bed nor to remove the lattice specimens from it.

**Figure 1:**
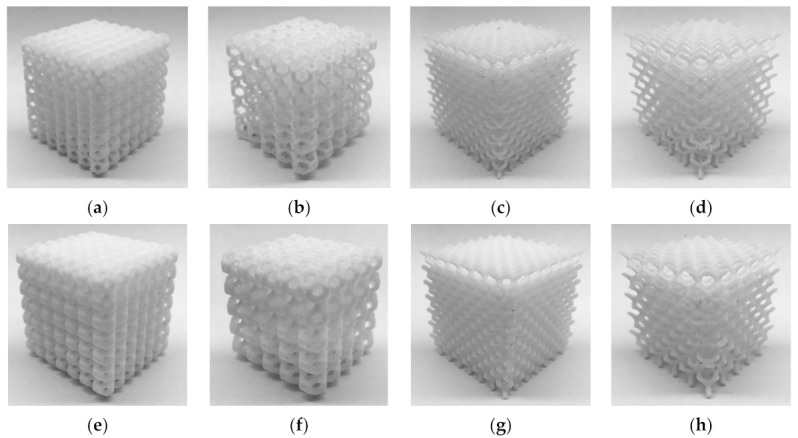
ABS printed specimens (**a**) 001; (**b**); 002; (**c**) 003; (**d**) 004; (**e**) 005; (**f**) 006; (**g**) 007; and (**h**) 008.

**Figure 2:**
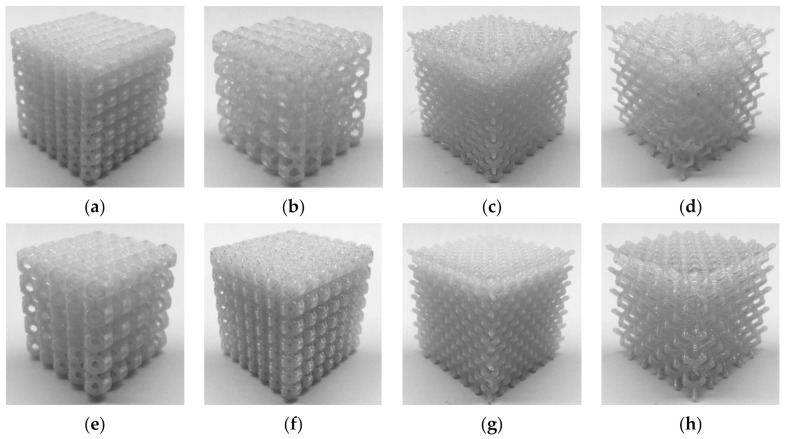
Polycarbonate (PC) printed specimens (**a**) 009; (**b**); 010; (**c**) 011; (**d**) 012; (**e**) 013; (**f**) 014; (**g**) 015; and (**h**) 016.

White ABS (Tiertime) and white PC (Flashforge) were used as feedstock materials. Mechanical properties are presented in Table 3. Table 4 shows the FDM parameters for both feedstock materials. Some parameters, such as temperature, nozzle diameter, and orientation were fixed for all the samples to focus on the influence of the parameters under study. The printing temperature was set according to the recommendations of the feedstock manufacturers, which gave satisfactory results in preliminary printing tests. Figure 1 and Figure 2 show some of the manufactured specimens in ABS and PC, respectively.

The printed specimens showed some differences when compared to the digital models. In addition to the geometrical variation of the strut related to the staircase effect, the effective diameter of struts increased. For struts with a nominal diameter of 1 mm, the as-printed strut diameter was in the range of 1.05–1.35 mm; for struts with a nominal diameter of 1.5 mm, the as-printed diameter was 1.55–1.75 mm. Nevertheless, the as-printed diameter might not necessarily represent the effective load-carrying diameter [30,36,37].

### 2.3. Compression Tests

A universal testing machine model WP 310 (50 kN, GUNT Gerätebau, Barsbüttel, Germany) with flat plates was used to carry out the compression tests. Specimens were tested in the same orientation of printing. The plates were 70 mm in diameter, thus limiting the size of the sample section to 35 mm × 35 mm. The preparation of the specimens and the compressive tests were carried out according to the standard test method for compressive properties of rigid cellular plastics, ASTM D1621 [48], with the number of specimens limited to two per case. A constant speed of 2 mm/min was used during the compression tests. No lubricant was used in the contact surfaces between specimens and plates. Compression tests were carried out until the samples were compressed to about 50% strain, as the densification phase was beyond the scope of this study.

Compressive stress (i.e., plateau stress) for all samples was determined from the experimental data. In this analysis, the plateau stress is defined as the arithmetical mean of stress values at 20% and 40% nominal strain, according to the standard for mechanical testing of cellular metals ISO 13314 [63].

## 3. Results

**Figure 3:**
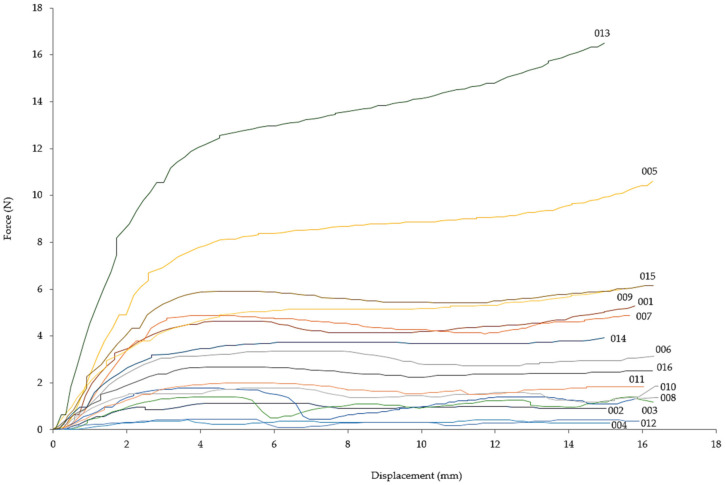
Force-displacement diagram of the compression tests for the 16 lattice configurations.

Figure 3 shows the force-displacement curves for compression tests of the 16 lattice configurations. The region of densification that commonly follows the plateau is not depicted, as the compression tests ended at around 45–50% nominal strain, before reaching densification strain. The broad range of curves reveals the overall effect of the variation of the parameters on their compressive response, with compressive forces ranging from 0.1 to 15 kN, although most lattices were in the range of 0.1–5.0 kN.

Mean values and standard deviations for the elastic modulus, plateau stress, and energy absorption capacity for the 16 lattice configurations are presented in Table 5. The energy absorption capacity quantified in Table 5 was measured as the area under the stress-strain curve up to a value of 40% nominal strain.

In some lattices, the standard deviation of the elastic modulus was noticeably larger, a consequence of small variations in the compressive force related to the non-homogeneous stress distribution inside the lattices during the test. To adjust for these oscillations, three measurements of the elastic modulus along different sections of the elastic region of the curve were made and then averaged.

Minitab 19 was used for the statistical analysis. Energy absorption capacity was not included in the analysis, as it is a direct outcome of the plateau stress.

**Figure 4:**
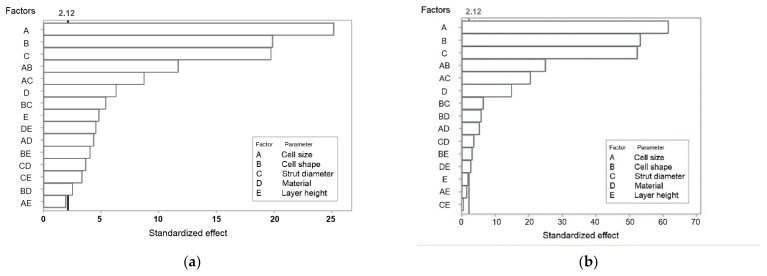
Pareto chart of standardized effects for (**a**) plateau stress, σ_pl_; (**b**) elastic modulus. The 2.12 line indicates a significance of the 95% confidence interval (α = 0.05).

Pareto plots were constructed for both plateau stress and elastic modulus (Figure 4). The bars that represent factors and interactions that cross the reference line at 2.12 are statistically significant at the 0.05 level. Both graphs show that factors A, B, and C, i.e., cell geometry parameters, are the most important factors defining the mechanical response. Interaction AB and AC are also important, showing a stronger influence than parameters D (feedstock material) and E (layer thickness).

**Figure 5:**
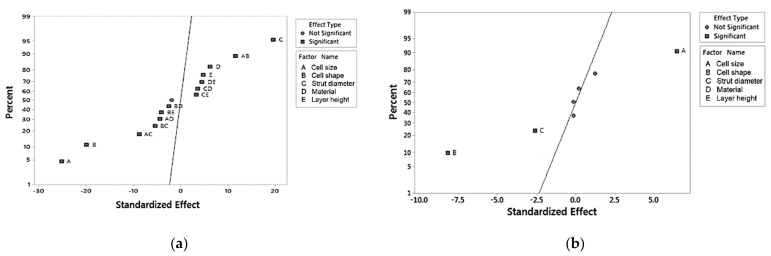
Normal plot of standardized effects for (**a**) plateau stress, σ_pl_; (**b**) elastic modulus, E. The 2.12 line indicates a significance of the 95% confidence interval (α = 0.05).

Figure 5 shows the normal probability plot of effects for plateau stress and elastic modulus. The plots depict the magnitude, direction, and importance of every factor and interaction: those effects that are further from 0 are statistically significant at the 0.05 level. For both plateau stress and elastic modulus, the main effects for all factors (A, B, C, D, and E) are statistically significant.

The plot also indicates the direction of the effect. Cell size (A) and cell shape/type (B) have a negative standardized effect, i.e., a change from the low level to the high level of the factor causes a decrease in response. On the other hand, factors C, D, and E have positive standardized effects, so a change from the low to the high in any of them increases the outcome values.

**Figure 6:**
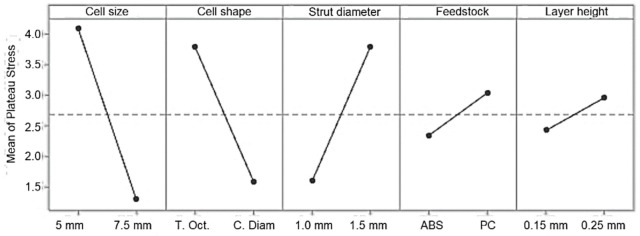
Main effect plots for plateau stress, σ_pl_.

**Figure 7:**
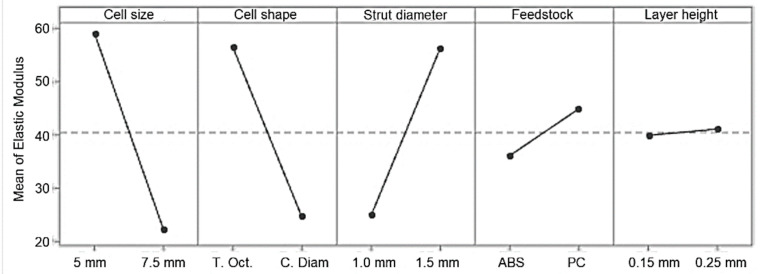
Main effect plots for elastic modulus, E.

Main effects plots are presented to compare the relative strength of the effects of the five factors on the plateau stress and elastic modulus (Figure 6 and Figure 7). Factors with a larger slope have a bigger impact on the mechanical properties. Thus, geometric parameters (cell shape/type, cell size and strut diameter) have the largest influence on plateau stress and elastic modulus, while the effect of feedstock material and layer thickness/height is much smaller. Factor A and B at the low level (cell size 5 mm, truncated octahedron) and C, D and E at the high level (strut thickness 1.5 mm, PC and layer height 0.25 mm) yield maximum mechanical properties. The main effect values for both plateau stress and elastic modulus are shown in Table 6 and Table 7.

Another way to describe the main and interaction effects for two-level designs is using a regression model [55]. Excluding the least significant parameters, a model for plateau stress was obtained

Plateau stress = 3.1327 − 1.7509 A − 1.5184 B + 1.2981 C + 0.4976 D + 0.3391 E + 0.9739 A × B − 0.6836 A × C − 0.3836 A × D − 0.1776 A × E − 0.4838 B × C − 0.2590 B × D
(1)

and similarly, a reduced model for elastic modulus

Elastic modulus = 40.439 − 18.395 A − 15.916 B + 15.656 C + 4.426 D + 0.599 E + 7.482 A × B − 6.133 A × C − 1.595 A × D + 0.481 D × E
(2)

Table 6 shows the coefficient values for the regression model for the plateau stress. All p values are 0.05 or less, confirming that all variables have some degree of influence on the response with a 95% confidence. Similarly, Table 7 shows the coefficient values for the regression model for the elastic modulus. The results were statistically significant for all factors (*p* < 0.05), except for layer height (*p* = 0.062), cell size × layer height (*p* = 0.126), and strut diameter × layer height (*p* = 0.595).

**Figure 8:**
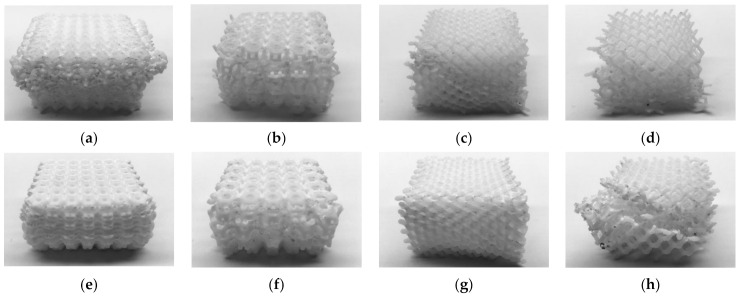
ABS printed and tested samples (**a**) 001; (**b**) 002; (**c**) 003; (**d**) 004; (**e**) 005; (**f**) 006; (**g**) 007; and (**h**) 008.

**Figure 9:**
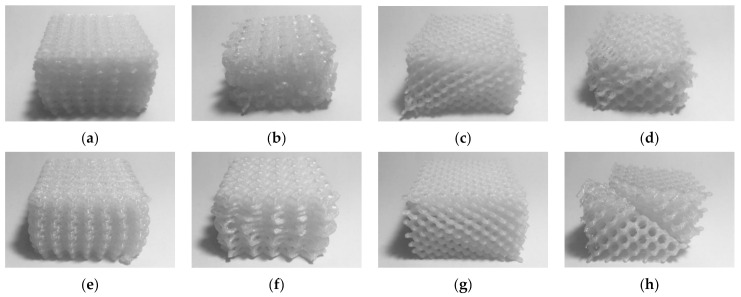
PC printed and tested samples (**a**) 009; (**b**) 010; (**c**) 011; (**d**) 012; (**e**) 013; (**f**) 014; (**g**) 015; and (**h**) 016.

Deformed specimens for every lattice are presented in Figure 8 and Figure 9. Barreling is noticeable in the compressed specimens of the two strongest lattice structures, 005 and 013 (Figure 8e and Figure 9e). The barreling could be attributed to the friction between specimens and the machine plates and is similar to the response of homogeneous materials. All specimens showed signs of damage localization. This behavior is consistent with the mechanical response of porous materials under compression, in which deformation is associated with strain localization, typically in the form of bands [64,65], which determine the onset of yielding, hardening, and the level of plateau stress [66].

In some cases, the localization was visible in the form of horizontal localization bands, i.e., crushed layers of cells (Figure 8a,b). In those samples, the plastic collapse of the structure took place on a layer-by-layer basis, with minimal (Figure 8b,e) or localized (Figure 8d) variations in its cross-section. In other materials, the localization took place along the diagonal of the specimen, leading in some cases to the fracture of the specimen (Figure 8a and Figure 9a). The width of the diagonal localization band was close to the cell size. Similar patterns were reported for octet-truss (or face-centered cubic) and truncated octahedron lattice materials manufactured by other AM methods [58,67,68].

## 4. Discussion

Although the manufacturing of small geometric features pushes FDM to its practical limits, variations in the mechanical response among the first and second samples of the same lattice were negligible in all but two cases (014 and 016), reflecting a good consistency in the manufacturing process. The two failed samples were discarded (according to standard ASTM D1621) and replaced by new specimens. While the new 016 specimen showed the expected behavior, the new sample of lattice 014 failed to reach the maximum value of plateau stress, as evidenced by the large standard deviation in plateau stress (Table 5). This scattering of mechanical properties was specific to lattice 014. A direct comparison with lattice 006, which has the same geometry as lattice 014, eliminates this as a possible explanation, which leads to assuming that the effect of manufacturing parameters (feedstock material and layer thickness)—or a superposition of multiple factors—could be the cause of the poor repeatability.

Some materials showed significant fluctuations in strength during the compressive test (Figure 3). In some cases, the fluctuation was present as a sudden decline, such as in lattices 003 (1.36 to 0.31 kN), 008 (1.36 to 0.45 kN), and 012 (0.45 to 0.09 kN). Although strain localization bands are visible in the compressed specimens (Figure 8c,h and Figure 9c,d), they are also visible in the other cubic diamond lattices that did not experience a sudden decline. Nevertheless, it is noteworthy that the reduction of strength arose when the displacement was about 5–7 mm, similar to the cell size in the lattices.

The best mechanical properties were obtained for specimens 013 and 005, with both structures having identical cell size (5 mm), unit cell type (truncated octahedron), and strut thickness (1.5 mm). On the other hand, the poorest mechanical response was obtained by structures 004 and 012, both having the same cell size (7 mm), unit cell type (cubic diamond), and strut thickness (1 mm).

The energy absorption capacity of lattices 005 and 013 was 4.25 MJ/m^3^ and 3.05 MJ/m^3^, respectively, which is significantly larger than values reported earlier for polymer lattice structures with larger cells [7]. The energy absorption capacity of specimens 005 and 013 was similar to that reported for titanium truncated octahedron lattices with over 80% porosity (1.6–6.5 MJ/m^3^) [58], thus evidencing potential for energy absorbing applications.

The influence of all five parameters on the compressive response of the cellular structures was statistically significant. The statistical analysis confirms that cell size and strut thickness influence the plateau stress of lattice structures. This outcome is consistent with previous studies that explored the influence of cell size and strut diameter on the mechanical response of lattices [7,28,29,30,31]. The strength and modulus of the lattices increased with the decrease in the unit cell size and/or increase in strut diameter, due to the associated denser structures [29]. Similarly, the analysis also confirms the results of previous studies that explored the mechanical response of both unit cell types [24,57,58]: truncated octahedron lattices showed better mechanical properties compared to cubic diamond lattices.

On the other hand, feedstock material and layer thickness played a secondary role, with PC and a larger layer thickness associated with better mechanical properties. This contravenes previous studies that reported a strong influence of layer thickness on the properties of FDM-fabricated solid parts [41]. Furthermore, this also suggests that the effect of strut irregularities, and its subsequent effect on the mechanical properties of miniature lattices, might not be greatly reduced by the minimization of the layer thickness.

The effect of feedstock material, although statistically significant, is secondary when compared to the geometrical parameters. Given that both the density and the moduli of both feedstock materials are similar (Table 1), a lesser effect on the elastic modulus was expected, as suggested by available scaling law models [22,28]. On the other hand, the compressive yield strength of solid PC doubles that of ABS, which should produce a large difference in plateau stress of lattice built using different feedstock material [22]. Nevertheless, the effect of feedstock on the plateau stress does not reflect this assumption. Given the wide variation in mechanical properties reported in the literature for these two materials [69], further analysis of this topic is necessary. Thus, additional compression tests under ASTM D695 were carried out for solid ABS and PC specimens built using FDM, showing similar yield stress values for both ABS and PC (40.99 MPa and 43.21 MPa, respectively).

Among the five parameters, layer thickness had the least influence on the mechanical response. Although layer thickness affects the strength and durability of solid FDM parts [41], its influence in FDM-fabricated lattices seems to play a secondary role. Given that manufacturing cost is directly related to layer thickness [43], it would be cost-effective to use the largest possible layer thickness. For instance, an increase in layer thickness from 0.15 to 0.25 mm would represent a 50% reduction in printing time.

The multilinear regression models (Equations (1) and (2)) could be used to determine the approximate variation of plateau stress and elastic modulus when one or multiple parameters are varied. Table 8 presents the variation in mechanical properties when single parameters are varied from high to low level. While a change in a single geometric parameter can produce a strong decrease in mechanical properties, a change in feedstock material or layer thickness causes a smaller effect. On the other hand, the influence of layer thickness on elastic modulus is marginal.

An analysis of variance was performed to assess the data and regression models. The *p*-values for all the main factors were statistically significant (0.05 or less), demonstrating that all parameters are relevant. The R^2^ value for both models is over 99.4%, which indicates that the models fit the data very well. The variance inflation factors are small, which evidenced that the factors were not correlated.

In contrast to analytical and numerical models, which overpredict the effective modulus [23], and semi-empirical methods based on the scaling law, which do not address geometric details of the lattices explicitly [9], the current approach could be used for the design of lattice structures with specific mechanical properties. Although limited in scope, the models are very accurate, and the parameters could be fine-tuned to obtain specific mechanical properties such as a set value elastic modulus, plateau stress, or even energy absorption capacity.

## 5. Conclusions

In this study, the effects on the compressive response of 3D printed miniature lattice structure of five factors were examined.

All tested parameters were found to have significant effects on the mechanical properties of the lattice structures. Cell geometry (i.e., unit cell type, cell size and strut diameter) was shown to have the largest effect on the mechanical response of the lattice structures, represented by the plateau stress and elastic modulus. On the other hand, feedstock material and layer thickness have significant effects on plateau stress, but its influence is minor when compared to the geometric parameters. Regarding the elastic modulus, layer height had no significant effect. This last observation goes against our current understanding of the effect of layer thickness on the mechanical properties of solid FDM fabricated parts.

Unit cell type, cell size, and strut diameter define the relative density, which determines the mechanical properties. Hence, a smaller cell size, a truncated octahedral cell, and thicker struts increase the density, and subsequently the mechanical properties of the cellular materials. Results also suggest that polymer truncated octahedron lattices have potential in protective applications, with an energy absorption capacity similar to that of high porosity metal foams, although further analysis is required.

Although the accuracy of the manufacturing process is limited, FDM could be a simple, cost-effective method for the fabrication of custom-made 3D lattice structures. Factorial design models, although limited in scope, could be an effective tool in the early stages of design of customized lattice structures.

## Figures and Tables

**Table 1:** Levels for the factorial analysis.

Parameter	Type	Low	High
Cell size	Quantitative	5 mm	7 mm
Cell shape/type	Qualitative	Truncated octahedron	Cubic diamond
Strut diameter	Quantitative	1 mm	1.5 mm
Feedstock material	Qualitative	ABS	PC
Layer thickness	Quantitative	0.15 mm	0.25 mm

**Table 2:** Mechanical properties of ABS [60,61] and PC [62].

Feedstock Material	ABS	PC
Density (g/cm^3^)	1.195	1.200
Elastic modulus (MPa)	2180	1958
Compressive yield strength (MPa)	30	64

**Table 3:** Designation of each specimen and its characteristics.

	Cell Size (mm)	Cell Shape/Type	Strut Diameter (mm)	Feedstock Material	Layer Height (mm)
001	5	Truncated octahedron	1.0	ABS	0.25
002	7	Truncated octahedron	1.0	ABS	0.15
003	5	Cubic diamond	1.0	ABS	0.15
004	7	Cubic diamond	1.0	ABS	0.25
005	5	Truncated octahedron	1.5	ABS	0.15
006	7	Truncated octahedron	1.5	ABS	0.25
007	5	Cubic diamond	1.5	ABS	0.25
008	7	Cubic diamond	1.5	ABS	0.15
009	5	Truncated octahedron	1.0	PC	0.15
010	7	Truncated octahedron	1.0	PC	0.25
011	5	Cubic diamond	1.0	PC	0.25
012	7	Cubic diamond	1.0	PC	0.15
013	5	Truncated octahedron	1.5	PC	0.25
014	7	Truncated octahedron	1.5	PC	0.15
015	5	Cubic diamond	1.5	PC	0.15
016	7	Cubic diamond	1.5	PC	0.25

**Table 4:** Printing parameters used for the manufacturing of samples.

Printing Parameters	ABS	PC
Extruder temperature (°C)	270	265
Bed temperature (°C)	90	90
Nozzle diameter (mm)	0.4	0.4
Extrusion width (mm)	0.35	0.35
Printing speed (mm/s)	undisclosed	undisclosed *
Support	No	No
Surface adhesion	Raft	Raft

* not reported by printer/software.

**Table 5:** Mean values and standard deviation for each lattice structure.

ID	Elastic Modulus (MPa)		Plateau Stress (MPa)		Energy Absorption Capacity (MJ/m^3^)	
	Mean	SD	Mean	SD	Mean	SD
001	63.43	4.72	4.17	0.03	1.55	0.04
002	15.48	0.30	1.00	0.05	0.32	0.07
003	19.65	2.33	1.10	0.01	0.39	0.00
004	4.49	0.10	0.28	0.02	0.09	0.01
005	89.62	0.66	7.82	0.21	3.06	0.02
006	42.27	1.34	2.68	0.03	1.10	0.15
007	57.57	3.00	3.08	0.02	1.12	0.12
008	18.21	2.47	1.11	0.01	0.32	0.01
009	60.42	4.55	4.79	0.03	1.81	0.02
010	20.39	2.48	1.52	0.03	0.59	0.03
011	21.19	1.39	1.55	0.07	0.55	0.01
012	3.05	1.02	0.29	0.01	0.08	0.00
013	130.66	0.22	12.84	0.13	4.27	0.16
014	33.72	1.90	2.48	0.93	1.09	0.05
015	59.83	0.68	3.88	0.06	1.95	0.81
016	25.60	4.62	1.54	0.01	0.54	0.05

**Table 6:** Regression coefficients for plateau stress.

Term	Effect	Coefficient	T-Value
Constant	-	3.1327	74.14
Cell size (A)	−3.5017	−1.7509	−41.44
Cell type (B)	−3.0367	−1.5184	−35.93
Strut diameter (C)	2.5961	1.2981	30.72
Feedstock Material (D)	0.9952	0.4976	11.78
Layer height (E)	0.6781	0.3391	8.02
Cell size × Cell type	1.9477	0.9739	23.05
Cell size × Strut diam.	−1.3672	−0.6836	−16.18
Cell size × Feedstock Mat.	−0.7672	−0.3836	−9.08
Cell size × Layer height	−0.3552	−0.1776	−4.20
Cell type × Strut diam.	−0.9677	−0.4838	−11.45
Cell type × Feedstock Mat.	−0.5180	−0.2590	−6.13
Cell type × Layer height	−0.6343	−0.3172	−7.51
Strut diam. × Feedstock Mat.	0.5881	0.2941	6.96
Strut diam. × Layer height	0.5958	0.2979	7.05
Feedstock Mat. × Layer height	0.8634	0.4317	10.22

**Table 7:** Regression coefficients for elastic modulus.

Term	Effect	Coefficient	T-Value
Constant	-	40.439	135.58
Cell size (A)	−36.790	−18.395	−61.67
Cell type (B)	−31.832	−15.916	−53.36
Strut diameter (C)	31.312	15.656	52.49
Feedstock Material (D)	8.851	4.426	14.84
Layer height (E)	1.198	0.599	2.01
Cell size × Cell type	14.963	7.482	25.08
Cell size × Strut diam.	−12.265	−6.133	−20.56
Cell size × Feedstock Mat.	−3.191	−1.595	−5.35
Cell size × Layer height	0.963	0.481	1.61
Cell type × Strut diam.	−3.921	−1.961	−6.57
Cell type × Feedstock Mat.	−3.496	−1.748	−5.86
Cell type × Layer height	−1.906	−0.953	−3.19
Strut diam. × Feedstock Mat.	2.211	1.105	3.71
Strut diam. × Layer height	0.324	0.162	0.54
Feedstock Mat. × Layer height	1.668	0.834	2.80

**Table 8:** Variation in mechanical properties when single factors are changed from the high to the low level.

Parameter	Plateau Stress	Elastic Modulus
Cell size	56%	42%
Cell shape/type	69%	61%
Strut diameter	43%	39%
Feedstock material	20%	11%
Layer thickness	9%	2%

## Data Availability

Data available upon request.
